# The perils of global signal regression for group comparisons: a case study of Autism Spectrum Disorders

**DOI:** 10.3389/fnhum.2013.00356

**Published:** 2013-07-12

**Authors:** Stephen J. Gotts, Ziad S. Saad, Hang Joon Jo, Gregory L. Wallace, Robert W. Cox, Alex Martin

**Affiliations:** ^1^Section on Cognitive Neuropsychology, Laboratory of Brain and Cognition, National Institute of Mental Health, National Institutes of HealthBethesda, MD, USA; ^2^Scientific and Statistical Computing Core, National Institute of Mental Health, National Institutes of HealthBethesda, MD, USA

**Keywords:** functional connectivity, typically developing, artifact, resting-state fMRI, GCOR, global correlation

## Abstract

We have previously argued from a theoretical basis that the standard practice of regression of the Global Signal from the fMRI time series in functional connectivity studies is ill advised, particularly when comparing groups of participants. Here, we demonstrate in resting-state data from participants with an Autism Spectrum Disorder and matched controls that these concerns are also well founded in real data. Using the prior theoretical work to formulate predictions, we show: (1) rather than simply altering the mean or range of correlation values amongst pairs of brain regions, Global Signal Regression systematically alters the rank ordering of values in addition to introducing negative values, (2) it leads to a reversal in the direction of group correlation differences relative to other preprocessing approaches, with a higher incidence of both long-range and local correlation differences that favor the Autism Spectrum Disorder group, (3) the strongest group differences under other preprocessing approaches are the ones most altered by Global Signal Regression, and (4) locations showing group differences no longer agree with those showing correlations with behavioral symptoms within the Autism Spectrum Disorder group. The correlation matrices of both participant groups under Global Signal Regression were well predicted by our previous mathematical analyses, demonstrating that there is nothing mysterious about these results. Finally, when independent physiological nuisance measures are lacking, we provide a simple alternative approach for assessing and lessening the influence of global correlations on group comparisons that replicates our previous findings. While this alternative performs less well for symptom correlations than our favored preprocessing approach that includes removal of independent physiological measures, it is preferable to the use of Global Signal Regression, which prevents unequivocal conclusions about the direction or location of group differences.

## Introduction

Interest in the functional organization of large-scale brain circuitry in normal and disordered populations has exploded in recent years. Out of the variety of methods and techniques in use to study this organization, much effort has been focused on studies of very slow fluctuations of brain activity during rest using BOLD fMRI (see Fox and Raichle, 2007, for review). In part, resting-state studies of inter-regional brain correlations, also referred to as “functional connectivity,” have proliferated because of the ease of acquiring the data. However, there are also promising potential benefits of the method for studying participant groups that are less able to perform complex behavioral tasks, including clinical populations (e.g., Fox and Greicius, 2010), human infants (e.g., Fransson et al., 2007), and animals (e.g., Vincent et al., 2007; Margulies et al., 2009).

The study of Autism Spectrum Disorders (ASD), in particular, has benefited from these methods, with a growing number of studies evaluating the hypothesis that the behavioral impairments in ASD result from abnormal brain connectivity (e.g., Castelli et al., 2002; Belmonte et al., 2004; Just et al., 2004; see Müller et al., 2011, for review). To date, most resting-state (as well as task-based) fMRI studies of ASD have found evidence of decreased correlations throughout a variety of brain regions involved in social processing (e.g., Kennedy and Courchesne, 2008; Monk et al., 2009; Assaf et al., 2010; Weng et al., 2010; Anderson et al., 2011b; Ebisch et al., 2011; Gotts et al., 2012; von dem Hagen et al., 2012). However, not all studies have found this pattern. A recent resting-state study with a relatively large participant sample (N ≈ 40 per group) reported a mixture of increased and decreased correlations in ASD relative to typically developing (TD) control participants (Rudie et al., 2013). Another recent study, given the well-publicized concern about residual head-motion artifacts in functional connectivity studies (e.g., Deen and Pelphrey, 2012; Power et al., 2012; Van Dijk et al., 2012), carefully examined head motion artifacts and failed to find large group differences in connectivity between ASD and TD participants (Tyszka et al., 2013).

Indeed, time-varying artifacts are a large source of concern in functional connectivity studies. Major sources of artifact include head motion (e.g., Power et al., 2012; Satterthwaite et al., 2012, 2013; Jo et al., 2013; Yan et al., 2013), non-neural physiological variation resulting from cardiac and respiration cycles (e.g., Glover et al., 2000; Birn et al., 2006, 2008; Shmueli et al., 2007; Chang and Glover, 2009; Chang et al., 2009), as well as hardware artifacts (e.g., Cordes et al., 2002; Jo et al., 2010). Much recent attention has been given in the literature to the confounding impact of head motion on group differences in correlation, while much less has been given to physiological and hardware artifacts, perhaps because many researchers still do not collect the independent cardiac and respiration measures and/or utilize the analysis tools that would permit more direct examination. The goal of preprocessing steps in resting-state fMRI studies is to remove as much nuisance or “noise” variation from the time series as possible in order to allow observed correlation patterns (and group differences) to reflect the underlying neural interactions rather than non-neural artifacts. Not all preprocessing recipes are as comprehensive or direct in addressing the myriad of noise sources as others, and there is no currently accepted standard in the field for these critical noise cleaning procedures. A principal difficulty is to remove noise/artifact components of the time series data without removing neurally-derived components.

The goal of the current paper is to draw attention to the detrimental effects of the still common practice of removing the Global Signal (GS), the average time series in a whole-brain mask, from the data prior to comparing groups of participants. Multiple motivations for including the GS as a nuisance regressor have been articulated, including that it helps to remove uninteresting global fluctuations that mask circuit-level organization, that it captures global physiological artifacts that other tissue-derived measures from the ventricles or white matter fail to capture, and that it enhances the strength and reliability of experimental results (e.g., Fox et al., 2009; Keller et al., 2013). Most recently, the GS has been argued to provide additional aid in attenuating residual motion artifacts that can confound group comparisons (Satterthwaite et al., 2013; Yan et al., 2013). However, including the GS as a nuisance regressor can also have a number of undesirable effects. Its role in introducing negative correlations that are otherwise largely absent from fMRI correlations has been widely discussed (Fox et al., 2009; Murphy et al., 2009; Anderson et al., 2011a). It has also been demonstrated in monkeys that the GS in fMRI is tightly coupled with electrical neural activity (local field potential recordings) across a range of frequencies (Schölvinck et al., 2010). Removing it will therefore be expected to alter the actual pattern of neural interactions that one desires to measure, a point recently acknowledged by some of the originators of the practice (Snyder and Raichle, 2012).

**Figure 1:**
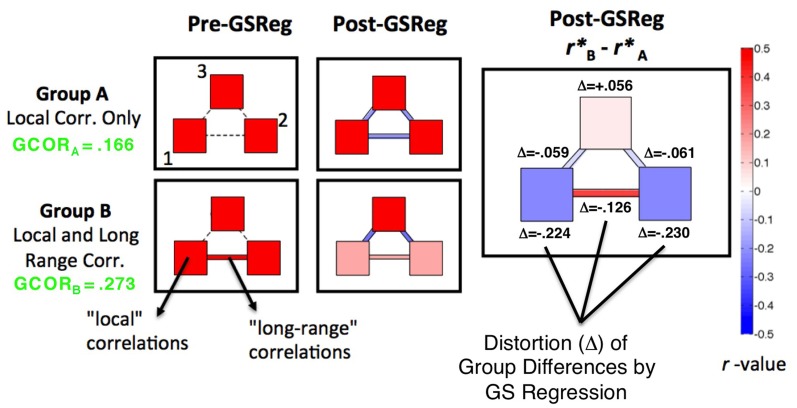
**Distortion of simulated group differences in correlation by GS regression**. Adapted from Figure 4 in Saad et al. (2012), patterns of correlation are shown for two simulated groups of participants, Group A and B (*N* = 30 in each). Pre-GS regression (**left panels**), both groups have three patches of simulated voxels (counter-clockwise from lower left: patches 1, 2, and 3) that have average within-patch correlations of 0.5 (see color bar to the right). Group B also has a correlation across patches 1 and 2, with all other inter-patch correlations in both groups set to be approximately 0. The presence of the across-patch correlation in Group B leads to an overall larger level of global correlation (GCOR values shown to left in green). After GS regression (**middle panels**), negative correlations are introduced among many of the patches and a larger amount of global variation is removed from patches 1 and 2 in Group B. Significant group correlation differences (**right panel**) are then found at all locations instead of at the one appropriate location (correlation between, not within, patches 1 and 2). The appropriate group differences are most distorted (Δ) by GS regression in and between patches 1 and 2, the locations involved in the largest true differences.

Less widely discussed to date are the detrimental effects for interpreting group comparisons. In a recent paper (Saad et al., 2012), we used simulation and mathematical analyses to show the impact of GS regression on correlation patterns and group comparisons, a summary of which is provided graphically in Figure 1. We simulated two groups of participants, A and B, for which the circuit-level structure differed in a simple way. In Group A, three simulated patches of voxels had positive correlations within but not across patches (correlations of zero). In Group B, correlations within patches were identical to A, with the only difference between groups being a positive correlation of 0.5 between patches 1 and 2. After GS regression (middle column of Figure 1), negative correlations were inappropriately introduced between patches for Group A, and the within-patch correlations were slightly reduced. For Group B, the presence of correlations among patches 1 and 2 led these time series to contribute relatively more to the GS than the time series in patch 3 (since they will weight into the global average more). Correspondingly, GS regression led more shared variation to be removed in patches 1 and 2, decreasing the related “local” and “long-range” correlations. In all, this procedure led to significant group differences being expressed at every location, rather than just at the single appropriate location (between patches 1 and 2) (rightmost column of Figure 1). The virtue of this demonstration is that the true statistics are known in all of their details, so it is clear that the effects one would observe after GS regression are artifactual. While the complexity of real data (and the absence of perfect knowledge about what patterns of data to expect) make these kinds of artifacts harder to examine, it is possible to derive three main predictions from this simulation and from a more comprehensive mathematical understanding of how GS regression should affect correlation matrices:

1. The equations that describe the influence of GS regression on any given correlation matrix show that the new matrix will depend in a complex manner on the initial matrix, with *all of the values* being “warped” to varying degrees (Saad et al., 2012, 2013). The prediction for real data is that rather than simply re-centering or re-scaling correlation values around a new mean value in an all-to-all matrix (0 after GS regression: Fox et al., 2009), the rank ordering of these values should also be altered. Furthermore, the alterations to the correlation matrix relative to the absence of GS regression can be predicted in a straightforward manner;
2. If two groups differ in their global level of correlation (as might be expected for ASD participants relative to TD participants), then the resultant re-centering of the corresponding correlation matrices to 0 after GS regression will necessarily lead to group differences in both directions and in locations where they should not occur, both in “local” correlations and in “long-range” correlations—even if the underlying group differences go in a single direction (see Jones et al., 2010, for an example of this in task-based functional connectivity of ASD). Note that the global level of correlation (GCOR), the grand average of the all-to-all correlation matrix, is lower in Group A than in Group B (green text in the left column of Figure 1);
3. Group comparisons will not be altered indiscriminately. They will tend to be altered most in locations that exhibit the largest underlying group differences - differences that are large enough and coherent enough over the spatial extent of the brain that they affect the GS measure. This is shown in the rightmost column of Figure 1, with the magnitudes of distortion (Δ) from the underlying group differences being the largest within and across patches 1 and 2. There is a simple mathematical explanation for this phenomenon. Whole-brain “connectedness” measures have been used in a number of studies to find locations of high connectivity with the rest of the brain (i.e., “hubs”: Buckner et al., 2009; Cole et al., 2010) and locations that differ between two groups in their interaction with the rest of the brain (e.g., Salomon et al., 2011; Gotts et al., 2012). The primary difference between whole-brain connectedness and correlation with the GS is simply whether whole-brain averaging is done before or after the correlation calculation. Indeed, if the time series are first transformed to *z*-scores with unit variance (allowing each voxel to contribute equally to the GS), whole-brain connectedness using Pearson correlation is directly proportional to both correlation and regression with the GS, with the effect of GS removal being greater removal of the largest connectedness differences.

In the remainder of this paper, we systematically vary the preprocessing procedures in order to evaluate these predictions in our own previously published ASD and TD resting-state data (Gotts et al., 2012). In addition to the GS and our preferred ANATICOR de-noising approach, which more explicitly models physiological and hardware artifacts (Jo et al., 2010), we evaluate a simple alternative to GS regression when independent cardiac and respiration measures are not available. The alternative, referred to as GCOR (for Global Correlation, Saad et al., 2013), treats the level of global correlation amongst all brain voxels as a nuisance covariate at the group-level of analysis, after the relevant correlation measures have already been calculated for each individual participant without the use of GS regression.

## Materials and methods

### Participants

The full details of our participant sample have already been published previously (Gotts et al., 2012). Twenty-nine typically developing (TD) participants (28 males, 1 female) between 12 and 23 years of age and 31 high-functioning participants (29 males, 2 females) with an autism spectrum disorder (ASD) between 12 and 23 years of age took part in the study. ASD participants were recruited from the Washington, DC metropolitan area, and all met *Diagnostic and Statistical Manual-IV* diagnostic criteria as assessed by an experienced clinician (20 Asperger's syndrome, 7 high-functioning autism, and 4 pervasive developmental disorder-not otherwise specified). Thirty ASD participants received the Autism Diagnostic Interview (ADI or ADI-R) (Le Couteur et al., 1989; Lord et al., 1994) and the Autism Diagnostic Observation Schedule (ADOS, Modules 3 or 4; Lord et al., 2000), administered by a trained, research-reliable clinician. All scores from participants with ASD met cut-off for the category designated as 'broad autism spectrum disorders' according to criteria established by the National Institute of Child Health and Human Development/National Institute on Deafness and Other Communication Disorders Collaborative Programs for Excellence in Autism (see Lainhart et al., 2006). Because the ADI and ADOS do not provide an algorithm for Asperger's syndrome, Lainhart and colleagues developed criteria that include an individual on the broad autism spectrum if s/he meets the ADI cut-off for “autism” in the social domain and at least one other domain or meets the ADOS cut-off for the combined social and communication score. Scores on the Social Responsiveness Scale (SRS) (Constantino, 2002), an informant-based rating scale used to assess ASD social and communication traits quantitatively over the full range of severity, were obtained from parents for 29 ASD participants. IQ scores were obtained for all participants, and all full-scale IQ scores were ≥ 85 as measured by the Wechsler Abbreviated Scale of Intelligence (26 ASD, 29 TD), the Wechsler Adult Intelligence Scale-III (3 ASD), or the Wechsler Intelligence Scale for Children-IV (2 ASD). Participant groups did not differ in terms of full-scale IQ, age, or sex ratio (see Gotts et al., 2012, Table 1). Informed assent and consent were obtained from all participants and/or their parent/guardian when appropriate in accordance with a National Institutes of Health Institutional Review Board-approved protocol.

### fMRI imaging methods

fMRI data were collected using a GE Signa 3 Tesla whole-body MRI scanner at the NIH Clinical Center NMR Research Facility using standard imaging procedures. For each participant, a high-resolution T_1_-weighted anatomical image (MPRAGE) was obtained (124 axial slices, 1.2-mm slice thickness, Field of View = 24 cm, 224 × 224 acquisition matrix). Spontaneous, slowly-fluctuating brain activity was measured during fMRI using a gradient-echo echo-planar series with whole-brain coverage while participants maintained fixation on a central cross and were instructed to lie still and rest quietly (*TR* = 3500 ms, *TE* = 27 ms, flip angle = 90°, 42 axial contiguous interleaved slices per volume, 3.0-mm slice thickness, FOV = 22 cm, 128 × 128 acquisition matrix, single-voxel volume = 1.7 × 1.7 × 3.0 mm). Each resting scan lasted 8 min and 10 s for a total of 140 consecutive whole-brain volumes. Independent measures of nuisance physiological variables (cardiac and respiration) were recorded during the resting scan for later removal in the majority of participants (24 ASD, 22 TD). Seven additional participants without these measures were included in each group after comparing descriptive statistics of the whole-brain-averaged EPI time series post-preprocessing to those calculated for the participants with measures present (see Gotts et al., 2012, Supplementary Materials and Methods, for full description). A GE 8-channel send-receive head coil was used for all scans, with a SENSE factor of 2 used to reduce gradient coil heating during the session.

### fMRI preprocessing

Four preprocessing models were compared in the current study. All preprocessing conditions utilized the AFNI software package (Cox, 1996) and had the following series of steps in common. The first 4 EPI volumes were removed from the resting scan, and large transients in the remaining volumes were removed by constraining values to be within 4 standard deviation units of the mean (using AFNI's 3dDespike). Volumes were then slice-time corrected, co-registered to the anatomical scan, resampled to 2.0-mm isotropic voxels, smoothed with an isometric 6-mm full width half maximum Gaussian kernel, normalized by the mean signal intensity in each voxel to reflect percent signal change, and transformed into the standardized Talairach and Tournoux (1988) volume for the purposes of group analyses. Tissue-based nuisance regressors were created by segmenting the anatomical scan into tissue compartments using Freesurfer (Fischl et al., 2002). Ventricle and white-matter masks were created, eroding the outer voxels of the masks to prevent partial volume effects with grey matter. Eroded masks were then applied to the volume-registered EPI data (prior to smoothing) in order to yield nuisance time series with minimal contribution from gray matter signals for the ventricles, as well as a local average, at each voxel, of the EPI signal from the (eroded mask) white matter voxels within a 15 mm radius of the central voxel.

#### Basic model: Motion + Ventricles + Local WM

The “basic model” is a reduced version of our full ANATICOR model without the independent physiological measures. It is common to the other three preprocessing models considered in this study. As indicated by the label above, nuisance variables for each voxel included the 6 head motion parameters (3 translation, 3 rotation) derived from the volume registration step, one average time series from the eroded ventricle mask, and the “local” average white matter time series. Throughout the remainder of the paper, the shorthand label “Basic” model refers exclusively to this preprocessing pipeline. The Basic model has two essential virtues that convey to the remaining preprocessing models: (1) it virtually eliminates the distance-dependent artifacts that result from transient head motion, even for the high movement cohorts such as the children cohort reported in Power et al. (2012) (Jo et al., 2013; see also Gotts et al., 2012, Supplementary Figures 5–11), and (2) the local white matter regressor (Local WM) markedly attenuates transient hardware artifacts that result from faulty channels in send/receive head coils and that generate spatially restricted signals in adjacent white and gray matter voxels (Jo et al., 2010). Indeed, TD participants from our study served as examples of the artifact in Jo et al. (2010). The EPI time series and all nuisance time series were detrended with fourth-order polynomials prior to least-squares model fitting to each voxel's time series. No further temporal filtering was applied to the Basic model, since cardiac and respiratory cycles (frequencies above the Nyquist frequency of 0.5 * 1/TR ≈ 0.14286 Hz) are aliased to lower frequencies, preventing a bandpass filter from removing them appropriately.

#### Basic Model + GCOR

The temporal preprocessing steps in the +GCOR model are identical to the Basic model. The only addition is the use of the Global Correlation (or GCOR) measure as a nuisance covariate in the group analyses, after the correlation values of interest have already been calculated. This is explained in full in the section fMRI Analyses.

#### Basic Model + GS regression

In the +GS Regression model, the GS has been added to the list of nuisance regressors in the Basic model. The GS is calculated by applying a whole-brain mask for each participant to the volume-registered EPI time series to yield one average time series. As with the other nuisance regressors and the BOLD time series, the GS was detrended with fourth-order polynomials prior to least-squares model fitting.

#### Anaticor

This is the preprocessing model used in our prior study (Gotts et al., 2012). It consists of the Basic model plus regressors for RETROICOR (Glover et al., 2000; estimated for slice time 0) and Respiration Volume Per Time (RVT) (Birn et al., 2008), created from independently acquired cardiac and respiration measures during the EPI scan (sampling rate 50 Hz). These physiological regressors are intended to estimate: (1) aliased cardiac and respiration cycles, and (2) slower, BOLD-like effects of respiration (end-tidal CO_2_) that are typically below 0.1 Hz. These influences are not small for data in the current study, accounting for approximately 10–20% of variance in the EPI/BOLD signal and leading to Type II statistical errors if they are not removed (Gotts et al., 2012, Supplementary Figure 1).

### fMRI analyses

In Gotts et al. (2012), we developed an analysis approach to identifying resting-state correlation differences between ASD and TD participants throughout the entire brain (see also Anderson et al., 2011b; Salomon et al., 2011). In the current paper, we adopt a mixture of approaches intended to illustrate the impact of preprocessing steps on correlation differences calculated between pairs of regions that are sampled throughout the brain, as well as to estimate correspondences with our previously reported results.

#### Large-scale sampling of whole-brain mask with 1880 ROIs

**Figure 2:**
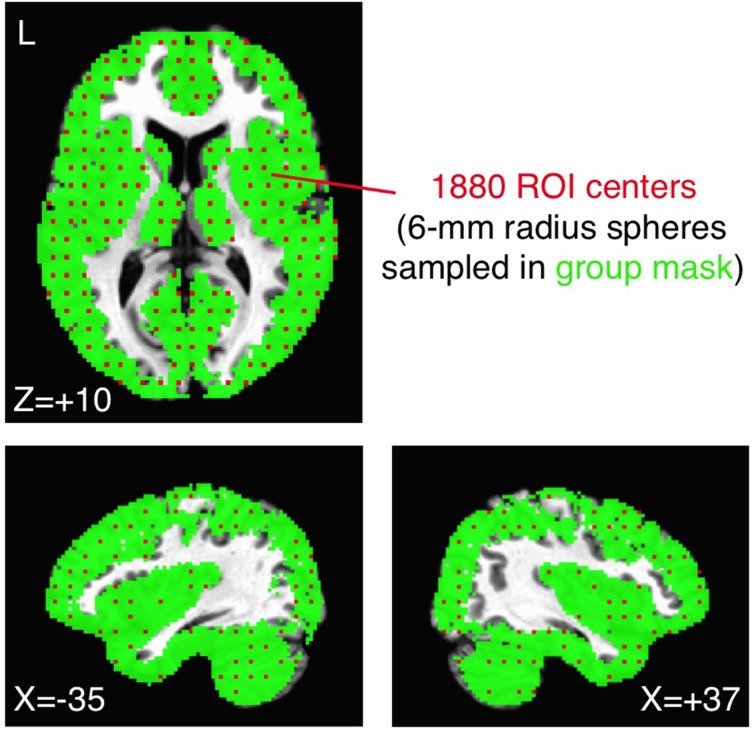
**Sampling the group brain mask with 1880 ROIs**. The original group brain mask from Gotts et al. (2012) (voxels shared in >85% of participants in both ASD and TD groups; shown in green) was sampled by choosing every fourth voxel from the original voxel grid (in X, Y, Z directions in Talairach coordinates). Each chosen voxel (red voxels) served as the center for a 6-mm radius sphere, totaling 1880 ROIs. The original group brain mask excluded voxels in white matter, the ventricles, and the sagittal sinus.

As one relatively comprehensive approach, we uniformly sampled spherical ROIs (6 mm radius) within our previous group brain mask (each voxel present in >85% of participants in each group). ROI centers were chosen by down-sampling the original voxel grid in Talairach coordinates to a new 4 × 4 × 4 grid of the original voxels (a new volume of 8 × 8 × 8 mm^3^), resulting in a total of 1880 ROIs (from 119,751 original voxels). Example ROI centers (each of which represents a 6 mm-radius sphere) are shown in Figure 2 in red, overlaid on the group brain mask in green. Note that the mask excludes ventricles, white matter, and the sagittal sinus, focusing on signals from the gray matter and subcortical structures. Correlations of the preprocessed average time series from each ROI for each participant were calculated in an all-to-all fashion and transformed to approximately normally distributed values (Fisher's *z* transform). Average group ROI-ROI correlation matrices were then calculated across participants within the ASD and TD groups and compared with two-sample *t*-tests. The relative rank ordering of correlation values within the ROI-ROI matrix was compared across preprocessing models using the Spearman rank correlation.

#### Assessing agreement with previous results using Gotts et al. (2012) ROIs

**Figure 3:**
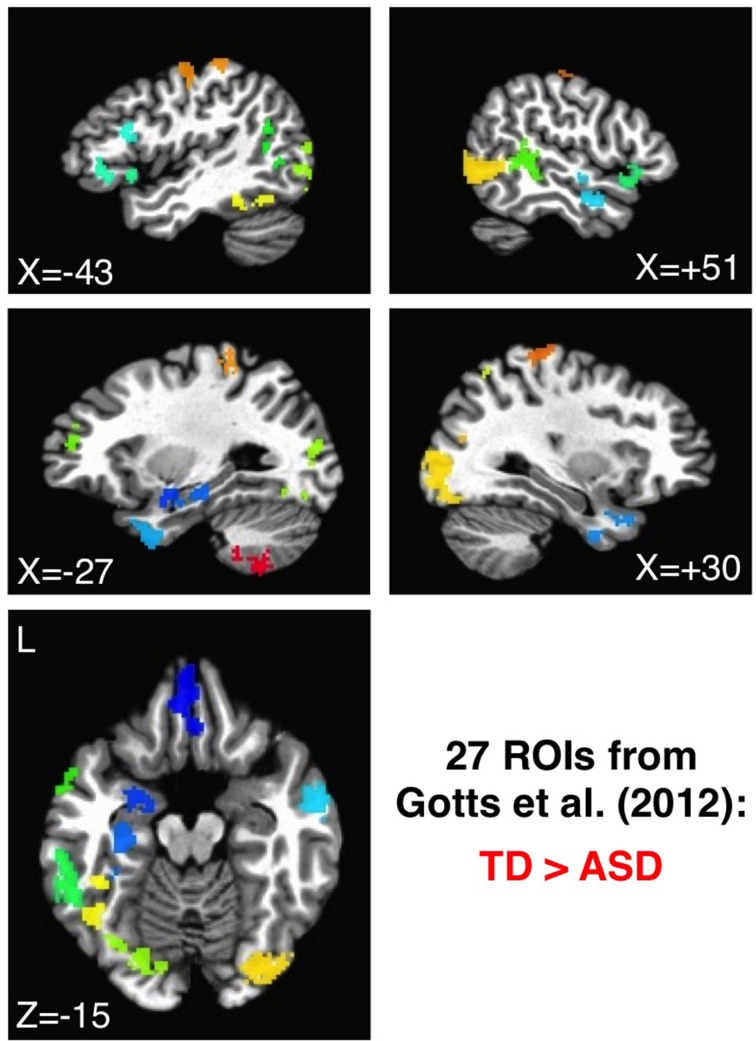
**ROIs showing the largest group differences (TD > ASD) in Gotts et al. (2012)**. ROIs 1–27 are shown using a distinct color for each ROI, ranging from cool colors (blue = 1) up to hot colors (red = 27).

The 27 ROIs identified in Gotts et al. (2012) as showing greater correlation in TD than in ASD participants were also applied to the de-noised data from each preprocessing model in order to evaluate consistency with our previously reported group comparisons, as well as with our previous ASD symptom correlations using the SRS total score (Constantino, 2002). These ROIs are shown in Figure 3, with each ROI assigned a unique color. As with the analyses using 1880 ROIs, the all-to-all ROI correlation matrix was calculated for each participant, comparing groups using two-sample *t*-tests after first transforming to normally distributed values (Fisher's *z*). In analyses of correlation with SRS total score, partial correlations were calculated across participants using the values in the ASD group at each ROI-ROI combination, removing the shared variation with Age and Full Scale IQ. Predictions regarding the influence of preprocessing model on “short-range” correlations were also assessed for these 27 ROIs. For these analyses, the average voxel-to-voxel Pearson correlation within each ROI was calculated for each ASD and TD participant, these values were then transformed using Fisher's *z*, and then they were compared across groups in each ROI using two-sample *t*-tests. The Pearson correlation was chosen for ease of implementation, and the results are not expected to depart markedly from those using canonical correlation and other similar methods (e.g., Regional Homogeneity or ”ReHo“: Zang et al., 2004; Paakki et al., 2010; Shukla et al., 2010; see also Jiang et al., 2013).

#### GCOR preprocessing model and analyses

**Figure 4:**
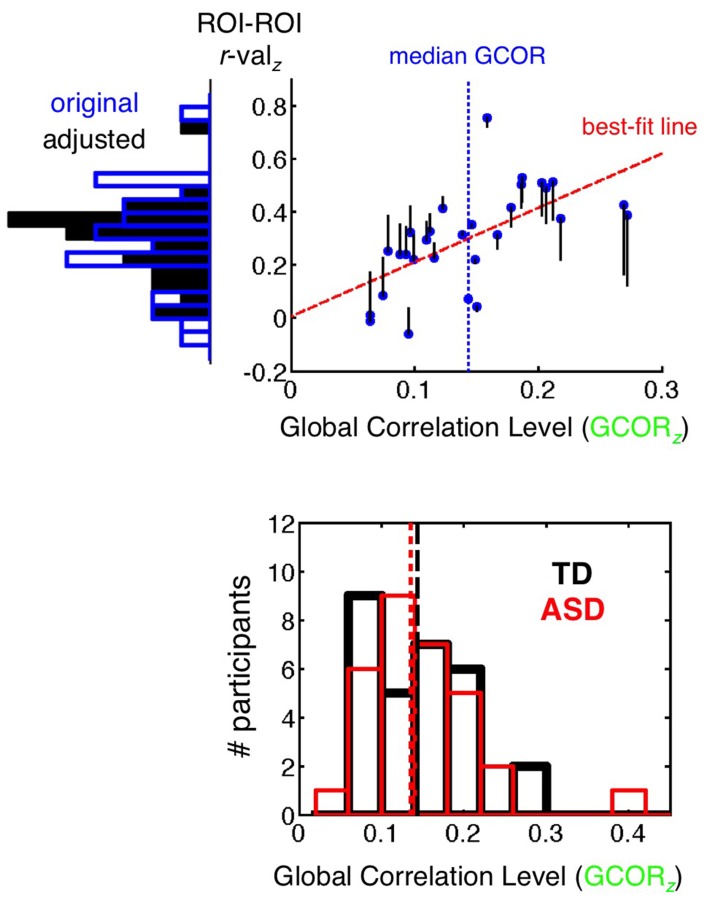
**GCOR method of removing global correlations. (top panel)** The *x*-axis shows the global level of Pearson correlation (GCOR) for each of the 29 TD participants, calculated among all possible voxel combinations in a whole brain mask and then transformed with Fisher's *z*. The *y*-axis shows the Fisher's *z*-transformed correlation value between two example ROIs for each participant, with frequency histograms across participants shown to the left of the *y*-axis. The blue dots are the original values of GCOR and ROI-ROI correlation for each participant under the Basic preprocessing model. Covariate removal is illustrated here for a single-group of participants, but appropriate removal for group comparisons is more complicated, carried out using Analysis of Covariance (ANCOVA), which is implemented in AFNI with the program 3dttest++ for two-level grouping variables. For a single group, the best-fit regression line (dashed red) is used to adjust y-values as a function of the distance from the median *x*-value (dashed blue vertical line). The adjusted values are shown relative to the blue dots using black vertical lines, with the new values at the endpoints. The adjusted values have a reduced standard deviation on the *y*-axis relative to the original distribution (see histogram of solid black bars on the left). (**bottom panel**) Frequency histograms of GCOR values are shown for TD (black) and ASD (red) participants. Distributions are overlapping and skewed for both groups, which motivated the choice of median rather than mean for re-centering.

The +GCOR model, as discussed above in the section Basic Model + GCOR, involves the same preprocessing steps as the Basic model. After the calculation of correlation coefficients between a pair of ROIs(/voxels) and the application of the Fisher's *z* transform, the GCOR method involves partialling out the influence of the global level of correlation (grand mean correlation of all voxels with all voxels in a whole-brain mask) on the group comparison of correlation values using an Analysis of Covariance (ANCOVA) approach (Saad et al., 2013). The top panel of Figure 4 provides a simplified illustration of the partialling process for a single participant group (the 29 TD participants) using an example pair of ROIs. The blue dots form a scatterplot of the Fisher's *z* GCOR value on the *x*-axis and the Fisher *z*-transformed ROI-ROI *r*-value on the *y*-axis across the TD participants. A frequency histogram of the *y*-axis values prior to GCOR removal (“original”) is shown to the left of the plot using blue-outlined bars. For this single-group example, the *y*-values are adjusted (vertical black lines leading away from the blue dots) using the slope of the best-fit line and the distance of the GCOR value from the group median GCOR value (shown with a vertical dashed blue line). The actual ANCOVA is more complex in implementation (program 3dttest++ in AFNI), involving the full model of grouping variable (2 levels: ASD and TD) and the continuous covariate (GCOR). The choice of mean or median for centering should depend on whether the distribution is approximately symmetrical or skewed, respectively (the GCOR distributions are skewed for both ASD and TD populations, shown in the bottom panel of Figure 4). The effect of covariate removal is to yield a more narrow distribution with reduced variance (frequency histogram of solid black bars to the left of the *y*-axis). This will tend to have the impact of increasing the amplitude of corresponding *t*-values when comparing groups if correlations in both groups strongly depend on the level of global correlation. For the analyses in the current paper, separate medians are used for centering each group, permitting differential levels of average correlation between the groups (as in Figure 1). If a single grand-mean or median is desired for centering both groups (depending on the study and hypotheses), then it is critical to verify that the groups being compared have similar overall ranges of GCOR. Otherwise, distortions similar to GS regression are expected to occur to a certain extent (see Saad et al., 2013, for further discussion).

#### Comparisons of whole-brain “connectedness”

In our prior study (Gotts et al., 2012), we compared functional connectivity levels between ASD and TD groups in a whole-brain manner by first finding the average correlation of each voxel with the rest of the brain mask (i.e., whole-brain “connectedness”; see also Salomon et al., 2011). Connectedness is similar, but not necessarily identical, to the measure of “degree centrality” in graph theory, and it is related to GCOR through calculation of a simple average over connectedness values. By comparing connectedness maps between groups, we identified good candidate “seeds” to be tested in subsequent analyses. We utilize this same whole-brain approach in the current study in order to identify the locations of strongest correlation differences between groups. Results for the Basic model and ANATICOR models were already presented in the prior study (see Gotts et al., 2012, Figures 2, 3, and Supplementary Figure 1). In the current paper, we conducted these analyses for the +GS regression and +GCOR models. The same statistical and cluster-size thresholds were used as in the prior study to afford direct comparisons of the preprocessing models (*p* < 0.05, uncorrected, with a spatial extent of at least 100 voxels).

#### Mathematical prediction of GS correlation matrices

Saad et al. (2012, 2013) have provided mathematical descriptions of the distortion in correlations induced by GS regression. In the current paper, we use these equations to predict the values of the correlation matrices for ASD and TD participants under the +GS preprocessing model using the time series data under the Basic model (without GS regression). These equations would be exact (i.e., equivalent to carrying out GS regression) if we were to use all voxel time series in a whole-brain mask. Here, we will use only data from the 1880 ROIs sampled from the group brain mask, excluding time series from white matter, ventricles and the sinuses. Therefore, the equations will only serve as predictive estimates, and these predictions will be accurate to the extent that the effects of GS regression depend primarily on gray matter signals and do not depend on signals in the excluded “non-neural” tissue compartments.

The equations used for these analyses are derived in detail in Saad et al. (2013), but we repeat them briefly here for convenience:

$$Z=\left(I-g{\left(g^{\text{T}}g\right)}^{-1}g^{\text{T}}\right)Y$$

where ***Z*** is the data matrix after GS regression (*N* time points x *M* voxels), ***I*** is the identity matrix, ***g*** is the GS of the *N*x*M* data matrix ***Y*** prior to GS regression. The time series in ***Y*** are presumed to have been de-meaned (i.e., means set to 0). Then:

$$\begin{array}{l} P=1/N\;Y^{\text{T}}Y\\ Q=1/N\;Z^{\text{T}}Z=P-\left(P11^{\text{T}}P\right)/(1^{\text{T}}P1)\\ R=P*\sigma_{P}\sigma_{P}^{\text{T}} \end{array}$$

where ***P*** and ***Q*** are the *M*x*M* covariance matrices of the ***Y*** and ***Z*** data matrices, ***R*** is the full correlation matrix based on ***Y***, * is the Hadamard element-wise matrix product, **σ**_*P*_ is the reciprocal square root (1/sqrt) of the diagonal elements (variances) of ***P***. Next,

$$\begin{array}{l} S=Q*\sigma_{Q}\sigma_{Q}^{\text{T}}\\ \;=\left(P-\left(P11^{\text{T}}P\right)/\left(1^{\text{T}}P1\right)\right)*\sigma_{Q}\sigma_{Q}^{\text{T}} \end{array}$$

where ***S*** is the correlation matrix after GS regression, ***1*** is an *M*x1 vector of ones, and **σ**_***Q***_ is the reciprocal square root of the diagonal elements of ***Q***. From this equation, it is clear that ***S*** is a function of the covariance matrix ***P*** of the data prior to GS regression. The “warping” effect of GS regression on the original correlation matrix ***R*** can then be seen by examining the difference ***S***-***R***:

$$S-R=\left(P-\left(P11^{\text{T}}P\right)/\left(1^{\text{T}}P1\right)\right)*\sigma_{Q}\sigma_{Q}^{\text{T}}-P*\sigma_{P}\sigma_{P}^{\text{T}}$$

This final equation shows that GS regression warps every value of the correlation matrix in a complex manner that depends solely on the covariance matrix ***P*** (the variance terms of ***Q*** are also dependent solely on ***P***).

For the purposes of the current analyses, we use the average time series calculated in the 1880 ROIs (Figure 2) under the Basic preprocessing model (without GS regression). This is tantamount to applying GS regression serially after the nuisance regressors in the Basic model have already been removed. This simplification will serve as a further potential source of inaccuracy in the estimation of ***S***, since the regression in the +GS model removes all nuisance variables simultaneously.

## Results

As discussed above, the main goal of the current paper is to evaluate three central theoretical predictions about the distorting effects of GS regression on group comparisons of functional connectivity in real data. To this end, we re-analyze resting-state data from 31 ASD and 29 TD participants that were originally reported in Gotts et al. (2012) using four different preprocessing models: (1) the Basic model (Motion + Ventricles + Local WM), (2) the Basic model +GCOR, (3) the Basic model +GS regression, and (4) our preferred ANATICOR model (Basic model + RETROICOR and RVT physiological regressors).

### Prediction 1: correlation matrices are “warped” under GS regression

**Figure 5:**
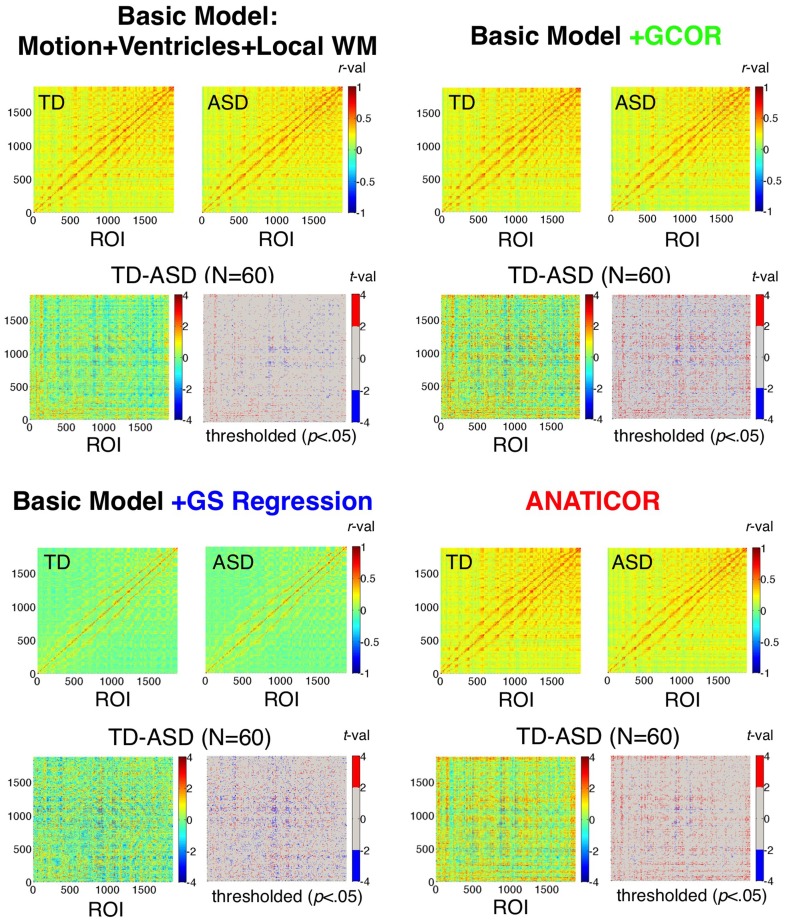
**Effect of preprocessing model on ROI-ROI correlations and group differences**. Correlation matrices for the TD and ASD groups and the corresponding group comparisons are shown for each of the four preprocessing models using 1880 ROIs sampled from the group brain mask. Results for the “Basic” model (Motion+Ventricles+Local WM) are shown in the upper left, the Basic+GCOR model in the upper right, the Basic+GS regression model in the lower left, and the full ANATICOR model in the lower right. The upper two plots of each model show the average ROI-ROI correlation matrices for the TD and ASD groups (see corresponding colorbars for scale), the lower left plot of each model shows the unthresholded *t*-values, and the lower right plot of each model shows the *t*-values thresholded at *p* < 0.05 (uncorrected). ROIs are ordered by scanner coordinates (ranked by Inferior-Superior, then by Anterior-Posterior, then by Right-Left).

We begin by evaluating the first prediction articulated in the introduction, namely that the effect of GS regression is not simply to re-center (alter the mean) or re-scale (alter the standard deviation) the correlations amongst a collection of voxel time series. Rather, the values are also “warped” as a function of the initial data covariance matrix, altering the rank orderings of the values within the all-to-all matrix. Correlations were calculated among all combinations of the 1880 ROIs (Figure 2) for the 31 ASD and 29 TD participants using the four preprocessing models. These results are shown averaged within each group in Figure 5 by preprocessing model. Also shown are the two-sample *t*-tests by ROI-ROI combination and thresholded *t*-maps (*p* < 0.05, uncorrected), with corresponding colorbars shown to the right of each plot. Few if any negative correlations were observed in either participant group for the Basic, +GCOR or ANATICOR models, whereas negative correlations were common in both groups under the +GS Regression model, yielding an average correlation value of approximately 0 for both groups. For both groups, the average correlation matrices are quite similar, both in scale and in rank-order for the Basic, +GCOR, and ANATICOR models. The grand mean (and standard deviation) of the correlation matrices for the ASD group were 0.2138 (0.1105), 0.2155 (0.1128), and 0.2028 (0.1111) for the Basic, +GCOR, and ANATICOR models, respectively. These same numbers for the TD group were 0.2222 (0.1133), 0.2275 (0.1160), 0.2240 (0.1166). Note that the TD group had an average correlation greater than the ASD group of approximately 0.01–0.02 across these three models. In contrast, for the +GS model the means (standard deviations) of the ASD and TD groups were 0.0155 (0.1165) and 0.0146 (0.1215), with the average correlation slightly larger for the ASD group. As with the mean correlations, the rank orderings of values within the ROI-ROI matrices were highly similar for the Basic, +GCOR, and ANATICOR models. Spearman rank correlations among these models were 0.9885 or larger for the ASD matrices and 0.9844 or larger for the TD matrices. In contrast, the Spearman rank correlation of the +GS model with the Basic, +GCOR, and ANATICOR models was 0.6896,0.6963, and 0.7125 for the ASD group and 0.6976,0.6930, and 0.6794 for the TD group. In other words, while better than 96.9% of the variance (*R*^2^ values) was shared in the rank orderings of group-average correlation values among the Basic, +GCOR, and ANATICOR models for both groups, approximately 50% of the variance was shared between the correlation matrices under the +GS model and those of the other models for both groups.

While it is difficult to compare these numbers statistically for the group-average matrices (there are statistical dependencies amongst the rows and columns), deriving the same measures for the ASD and TD individuals allowed comparisons and assessment of reliability across participants. Paired *t*-tests across participants within both the ASD and TD groups showed that the Spearman rank correlations of the +GS correlation matrices with the Basic and ANATICOR models were significantly reduced relative to the Spearman rank correlations between the Basic and ANATICOR models (+GCOR is applicable only to group-level analyses and was not part of these analyses). For the 31 ASD participants, average Spearman rank correlations of the +GS model with Basic and ANATICOR models were 0.7535 and 0.7261, respectively, whereas the average rank correlation between the Basic and ANATICOR models was 0.9362 [paired *t*_(30)_ > 8.50, *p* < 1.0e-08, for both; Bonferroni-corrected *P*-value = 0.05/3 = 0.0167]. For the 29 TD participants, Spearman rank correlations of the +GS model with the Basic and ANATICOR models were 0.7494 and 0.6971, respectively, whereas the rank correlations between the Basic and ANATICOR models was 0.9329 [paired *t*_(28)_ > 8.61, *p* < 1.0e-08, for both]. In summary, the rank ordering of the ROI-ROI correlation values for both ASD and TD participants is significantly altered or “warped” by GS regression, consistent with Prediction 1.

### Prediction 2: GS regression will alter the direction of group comparisons

The second prediction articulated in the introduction is that GS regression should alter the direction of group comparisons. In the case of Autism Spectrum Disorders, the prediction is that GS regression should lead to a higher incidence of ROI-ROI pairs for which ASD correlations are greater than TD correlations. This can occur for at least two reasons in the current context. First, if the average level of correlation differs between groups prior to GS regression (as shown in the previous section: TD > ASD), the re-centering of the average correlation (to approximately 0) will be differential in magnitude for the two groups, with a larger subtraction of correlation values from the TD group than from the ASD group. This will necessarily lead to reverse group differences (with ASD > TD) in some locations that did not differ prior to GS regression (possible Type I errors). Differential re-centering should also lead to the attenuation of real group differences in locations where they should be found (possible Type II errors). The second reason that correlation differences can become reversed after GS regression has to do with differential warping of the correlations in the two groups. A clear example of this phenomenon is shown in Figure 1, where larger shared variation is removed from patches 1 and 2 in Group B after GS regression compared to Group A. In either case, the expectation for real data is that the incidence of ASD > TD group differences should increase for the +GS model relative to the other models. For a previous task-based study (verbal fluency) of functional connectivity of ASD and TD participants in our lab, this phenomenon has already been demonstrated (Jones et al., 2010). In this section, we evaluate the effects of preprocessing model on the warping of the entire matrix of *t*-values, as well as on the relative incidence of significant group differences in both directions (TD > ASD and ASD > TD).

#### Warping of group comparisons by GS regression

**Figure 6:**
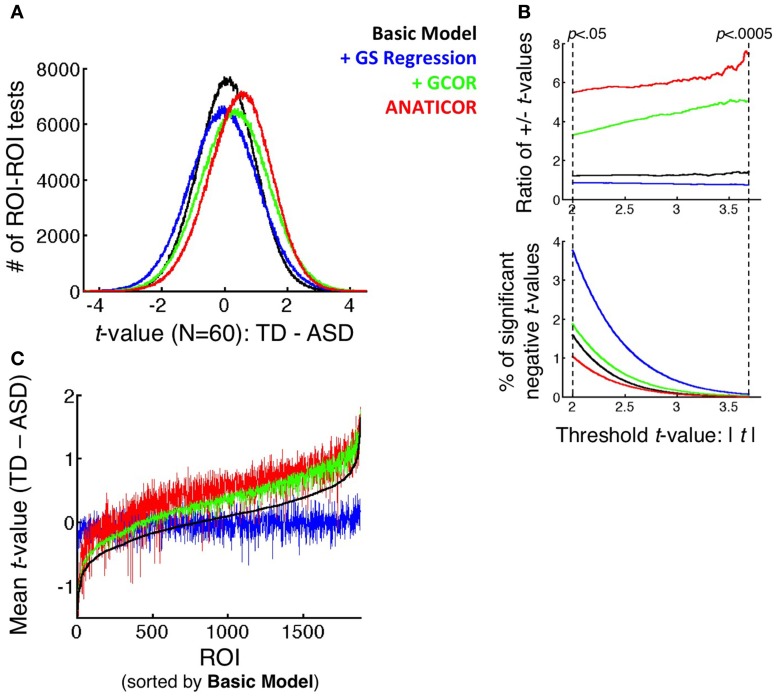
**Effect of preprocessing model on the distributions of group differences. (A)** Full distributions of *t*-values (TD-ASD) over all unique combinations of the 1880 ROIs (*N* = 1766260) under all four preprocessing models. **(B)** (top panel) Ratio of positive to negative *t*-values that survive the threshold *t*-value, shown as a function of the threshold on the *x*-axis (ranging from *p* < 0.05 to *p* < 0.0005, uncorrected). (bottom panel) Percentage of tests that yield significant negative *t*-values (i.e., favoring the ASD group) as a function of threshold *t*-value and preprocessing model. These values serve as the denominator in the ratios of the top panel. **(C)** Mean *t*-value across all 1880 ROI for each ROI as the seed (i.e., averaging across the rows of the full, unthresholded *t*-matrix of each model), rank-ordered from small to large by the mean *t*-values in the Basic model. These curves demonstrate that the largest alterations to the group comparisons by GS regression are for ROIs that elicit the largest average *t*-values under the +GCOR and ANATICOR models.

As with the average group correlation values for the ASD and TD groups among the 1880 ROIs (section Prediction 1: Correlation matrices are “warped” under GS regression), it was possible to evaluate the alteration of the corresponding *t*-values by preprocessing model. The means (standard deviations) of the *t*-values of the Basic, +GCOR, +GS regression, and ANATICOR models were 0.0593 (0.9438), 0.3127 (1.1015), −0.0327 (1.1015), and 0.4606 (1.0081) (see Figure 5 and summary histograms in Figure 6A). The Spearman rank correlations for the *t*-values among the Basic, +GCOR, and ANATICOR models were 0.8989 and greater (Basic with +GCOR:0.9899; Basic with ANATICOR: 0.8989; +GCOR with ANATICOR:0.9057). In contrast, the Spearman rank correlations of the +GS model with the others were 0.7504, 0.7604, and 0.6662 with the Basic, +GCOR, and ANATICOR models. In summary, the contrast *t*-values were most positive under the +GCOR and ANATICOR models, slightly positive for the Basic model, and slightly negative for the +GS model (i.e., greater correlations for the ASD participants). The rank orderings of the *t*-values were similar for the Basic, +GCOR, and ANATICOR models, despite differences in the mean values, sharing at least 80% of the variance among any combination of these models. In contrast, the rank orderings under the +GS model shared between 44 and 58% of the variance with those under the remaining models. All of these distributions are highly discriminable from one another, with *t*-values from paired *t*-tests well above 100 for all comparisons due to the diminishing standard error values for these very large sample sizes (*N* = 1766260 values in each).

#### Incidence of group differences in both directions as a function of preprocessing model

Both differential re-centering and warping of the correlation values by participant group predict a relatively higher incidence of group differences favoring the ASD group under the +GS model (the other models favor the TD group to varying degrees). Information about the relative likelihood of TD > ASD and ASD > TD group differences is present in graphical form in Figures 5, 6. The full matrices of *t*-values in Figure 5 show that the +GS model yields the most blue colors, indicating greater correlations for the ASD group. This is apparent both in the unthresholded and thresholded plots (lower left and right for each of the four models). In contrast, the +GCOR and ANATICOR models yielded the most positive (yellow/red) *t*-values, and the Basic model yielded fewer significant values in either direction (upper left panels of Figure 5). This can be quantified by counting the number of significant positive and negative *t*-values for a given significance threshold. Using *p* < 0.05 (uncorrected), out of 1766260 unique ROI-ROI combinations (1880 ROIs), the Basic model yielded 1.9155% significant positive *t*'s and 1.5759% negative *t*'s (+/−ratio: 1.2154), the +GCOR model yielded 6.1231% positive *t*'s and 1.8582% negative *t*'s (+/−ratio: 3.2952), the ANATICOR model yielded 5.6807% positive *t*'s and 1.035% negative *t*'s (+/−ratio: 5.4888). In contrast, the +GS model yielded 3.2192% positive *t*'s and 3.7507% negative *t*'s (+/−ratio: 0.8583). As the *P*-value threshold is lowered (down to *p* < 0.0005), ratios of positive to negative counts increase slightly for the +GCOR and ANATICOR models whereas they decrease for the +GS model (see Figure 6B). Combined with the information from section Warping of group comparisons by GS regression that the overall distributions of *t*-values are significantly shifted to more negative values for the +GS model relative to the other three models, it is clear that Prediction 2 (greater incidence of ASD > TD group differences) holds for this dataset across choice of statistical threshold (see also Jones et al., 2010). Indeed, the average TD-ASD *t*-value over all ROI-ROI combinations is significantly less than 0 under GS regression, a notable departure from the other models [mean = −0.0327, median = −0.0301, *SD* = 1.1015; one-sample *t*-test: *t*_(1766259)_ = −39.44, *p* < 1.0e-10].

### Prediction 3: GS regression will most alter the strongest group differences under other preprocessing models

The third prediction articulated in the introduction is that GS regression will not alter group comparisons indiscriminately. Rather, it will tend to alter results most in locations that exhibit the largest underlying group differences. One relatively simple way to evaluate this prediction for the current dataset is to first find regions out of the 1880 that yield the largest average group differences. This was done by averaging the *t*-values across the rows of the 1880 × 1880 *t*-matrices in Figure 5 for each preprocessing model. Then, these column-averaged *t*-values can be rank ordered from smallest to largest. Given the results in the section on Prediction 2, one expects the rank orderings of the Basic, +GCOR, and ANATICOR models to have quite similar rank orderings, whereas the +GS model should differ—at least relatively—in its rank orderings from these models. The critical prediction is that the ROIs with the largest average *t*-values for the Basic, +GCOR, and ANATICOR models should be the most altered in value for the +GS model. Rather than rank ordering the average *t*-values for each model separately (which would make it difficult to evaluate the agreement of particular ROIs in the rank ordering across models), we chose to rank order the ROIs relative to a single reference model, in this case the Basic model that is common to all of the other models. Figure 6C shows that the ROIs with the largest average *t*-values are quite similar for the Basic, +GCOR, and ANATICOR models (the black, green, and red curves, respectively). Indeed, the Spearman rank correlations of these 1880 column-averaged values ranged between 0.8848 and 0.9863 for these three models. In contrast, the average *t*-values of the +GS model are relatively flat when sorted by the *t*-values of the Basic model, indicating a strong alteration in the rank ordering. Accordingly, the Spearman rank correlation of the +GS model with the Basic, +GCOR, and ANATICOR models is 0.255, 0.2678, and 0.2623 respectively. Visually, it is clear from Figure 6C that the average *t*-values under the +GS model are most different from the +GCOR and ANATICOR models at the highest average *t*-values. In order to evaluate this phenomenon statistically, we compared the slopes of the best-fit regression lines to these curves. The slopes of the best-fit lines to the +GCOR and ANATICOR curves were 8.1509e-04 and 7.3080e-04, respectively, while the best-fit slope to the +GS curve was 0.73161e-04. The 99% confidence intervals calculated for the slope estimates were non-overlapping for the +GS model and those of the +GCOR and ANATICOR curves, demonstrating that they are significantly different from each other. The larger positive slopes for the +GCOR and ANATICOR models guarantees that they will differ most from the +GS model at their largest *t*-values.

### Mathematical prediction of ASD and TD correlation matrices under the +GS model

**Figure 7:**
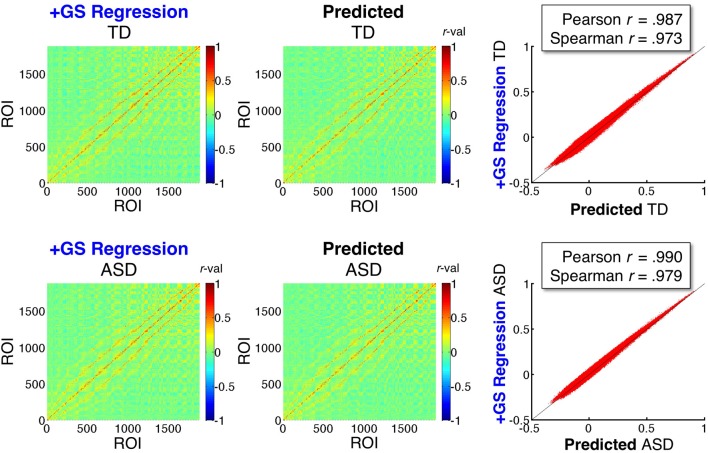
**Mathematical prediction of correlation matrices under GS regression**. The left two panels show the group average ROI-ROI correlation matrices for the TD and ASD groups under the +GS preprocessing model (shown also in Figure 5). The middle two panels show the matrices predicted by the equations developed by Saad et al. (2013) when applied to the time series data under the Basic preprocessing model (for equations used, see section Mathematical prediction of GS correlation matrices). Scatterplots of the agreement between the left and middle panels are shown in the rightmost panels, with Pearson and Spearman rank correlations shown to quantify the level of agreement. The predictions are accurate despite only estimating the distortions under GS regression from 1880 ROIs sampled in the group brain mask (excluding nuisance tissue signals such as white matter, ventricles, and sinuses).

In the sections above, we evaluated and confirmed three main predictions about effect of GS regression on group comparisons in real data. The purpose of the current section is to evaluate the extent to which the distorting effect of GS regression on a matrix of correlation values is captured by the equations of Saad et al. (2012, 2013). As described in section Mathematical prediction of GS correlation matrices, if we were to use all voxel time series in a whole brain mask, these equations would be exact (i.e., equivalent to performing GS regression). What makes this analysis more interesting is that only time series from the 1880 ROIs sampled from the group brain mask (Figure 2) were used for the estimation. Since the group brain mask excluded the brain tissue types that have been argued to contain the largest global nuisance signals (white matter, ventricles, and sinuses), successful prediction of +GS model correlations using only data from the Basic preprocessing model in these sampled ROIs would demonstrate that the main distorting effects of GS regression derive from averaging signals in gray matter voxels. Successful prediction will also highlight the fact that the equations describe the warping effect of GS regression correctly and that the reported distortions of group comparisons should not come as a surprise. Figure 7 shows the group-average ROI-ROI correlation matrices under the +GS preprocessing model for the ASD and TD groups in the left column and the matrices predicted from the sampled ROIs using the Basic model and the equations from Saad et al. (2013) in the middle column. Both Pearson correlation and Spearman rank correlations (scatterplots in right column) reveal that approximately 95% or better of the variation (*R*^2^ values) in the actual matrices are captured by the predicted matrices for both participant groups. These results indicate excellent performance of the equations, despite using only a subset of the voxel time series that were concentrated in gray matter. Note further that the agreement of the actual and predicted matrices is substantially higher than that between the +GS model correlation matrices and those under the other three models (approximately 50–55% of the variance shared).

### Anatomical locations of the strongest group differences under the +GS versus +GCOR models

**Figure 8:**
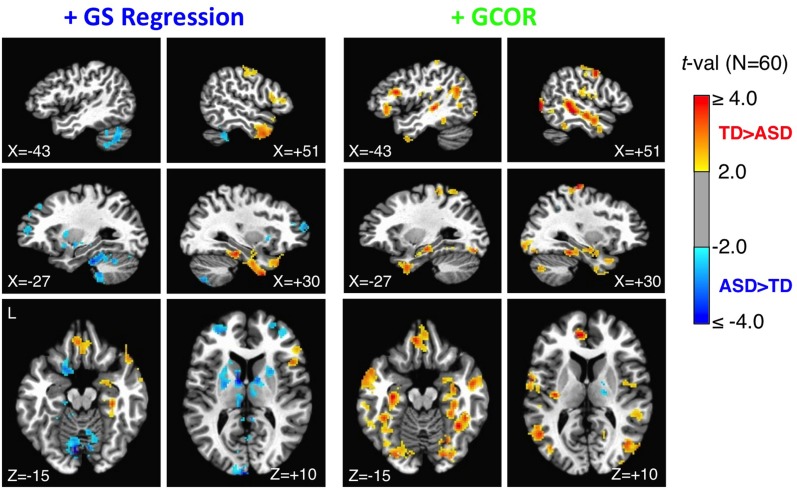
**Group comparisons of whole-brain connectedness for the +GS and +GCOR preprocessing models**. Whole-brain connectedness (i.e., the average correlation of each voxel time series with the rest of the voxels in the brain mask) was compared separately for the +GS regression and the +GCOR models. The +GS regression model, shown in the left plots, led to a larger number of locations with ASD connectedness values larger than TD values, as well as the absence of TD > ASD effects in locations found previously using ANATICOR. In contrast, the +GCOR method of removing global correlations, shown in the right plots, largely replicated the results found with ANATICOR (compare to ROIs in Figure 3 from the same sagittal and axial views). See text for full description.

Under one method of correcting for global correlations, GS regression, correlation matrices and group comparisons are distorted. Under another, GCOR, results appear to be qualitatively similar in many respects to our previously published results using ANATICOR. In order to facilitate more direct comparisons with the anatomical locations of our previous results in Gotts et al. (2012) (the 27 ROIs shown in Figure 3), we calculated whole-brain connectedness measures for each participant using both the +GS and +GCOR models and compared across groups using two-sample *t*-tests (see also Salomon et al., 2011). Using the same statistical and cluster-size thresholds as in the previous study, the results are shown for both models in Figure 8. The results for the +GCOR model are in good accord with our previous results, with greater connectedness values in the TD relative to the ASD group being observed throughout social brain areas, particularly in limbic-related brain regions (compare to Figure 3). In contrast, the +GS model yielded many more locations for which connectedness values were greater for the ASD group (note the results in cerebellum and striatum) and with relatively weak overall agreement with either the +GCOR or ANATICOR results.

### Effect of GS regression and other preprocessing models on “local” correlations

**Figure 9:**
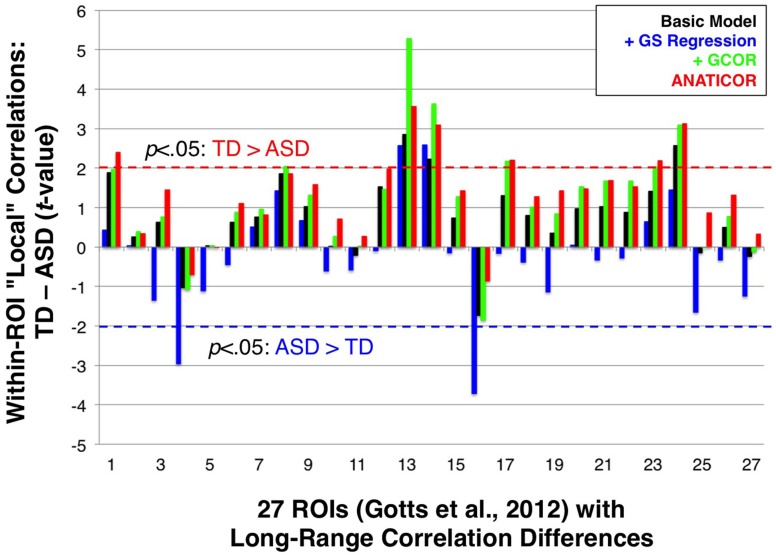
**Effect of preprocessing model on group comparisons of local correlation**. Group *t*-tests of local correlation (TD-ASD) under the four preprocessing models are shown for regions in Gotts et al. (2012) that exhibited greater long-range correlations for TD participants (ROIs 1-27; see Figure 3). Dashed red (TD > ASD) and blue horizontal lines (ASD > TD) mark the *p* < 0.05 significance level for individual tests. On average, the +GS model yielded more negative *t*-values (favoring the ASD participants) relative to the other three models.

In Figure 1, we highlighted the inter-dependence of long- and short-range correlations under GS regression. “Long-range” differences between groups that are large enough to manifest in the GS measure will have a tendency to be aliased into the “short-range” correlations involving the same voxels, although in the opposite direction as the long-range differences (note the reverse within-patch group differences in patches 1 and 2 after GS regression). In the current paper, we evaluated whether this same phenomenon occurs in our ASD/TD data by calculating “local” correlations among voxel time series within each of the 27 ROIs that we have shown exhibit greater long-range correlations for the TD group (Gotts et al., 2012; see Figure 3). If the groups exhibited equal levels of local correlation prior to GS regression, then there should be a tendency for significantly greater local correlations in the ASD group after GS regression. If the TD group exhibits larger local correlations prior to GS regression, then these differences should be attenuated or reversed after GS regression. In the event that the ASD group exhibits greater local correlations than the TD group prior to GS regression, then these differences should become enhanced after GS regression. In summary, since the long-range differences in these 27 ROIs favor the TD group, the influence of GS regression should be to shift the local correlations in these regions toward favoring the ASD group regardless of the initial direction of these differences. The results for the four preprocessing models, shown in Figure 9, are presented left-to-right from ROIs 1 to 27 (listed in the same order as Table 1 from Gotts et al., 2012). The Basic, +GCOR, and ANATICOR models all show a tendency for greater local correlations in the TD group, with results significant at *p* < 0.05 for 3, 6, and 6 ROIs out of the 27, respectively, and no ROIs showing significant differences favoring the ASD group. In contrast, the +GS model yielded results in 17/27 ROIs that numerically favored the ASD group (compared to 5, 4, and 3 out of 27 for the Basic, +GCOR, and ANATICOR models), with 2/27 ROIs showing significant differences (ROIs 4 and 16: the right ventromedial anterior temporal ROI and the left anterior superior frontal ROI). The +GS model also yielded results favoring the TD group in 2/27 ROIs, although with smaller *t*-values than for the +GCOR and ANATICOR models. The distributions of these *t*-values across ROIs did not differ from normality for any of the models, permitting their comparison with *t*-tests. The only two models that failed to show significant differences with each other are the +GCOR and ANATICOR models (*p* < 0.1). The +GS model yielded *t*-values that were significantly more negative than all of the other models [vs. Basic: paired *t*_(26)_ = −8.7585, *p* < 3.1055e-09; vs. +GCOR: paired *t*_(26)_ = −13.2229, *p* < 4.7379e-13; vs. ANATICOR: paired *t*_(26)_ = −11.8368, *p* < 5.6743e-12]. In accordance with the predictions articulated above, these results establish that the same aliasing of long-range correlation differences into reversed local correlations (as in Figure 1) occurs in our ASD/TD data. They also provide additional new evidence that local correlation differences between ASD and TD participants have a tendency to occur in the same direction as the long-range correlation differences when GS regression is not applied (i.e., favoring the TD participants; e.g., Khan et al., 2013; for further discussion, see Belmonte et al., 2004; Müller et al., 2011).

### Anatomical alignment of group differences and correlations with ASD social symptoms

**Figure 10:**
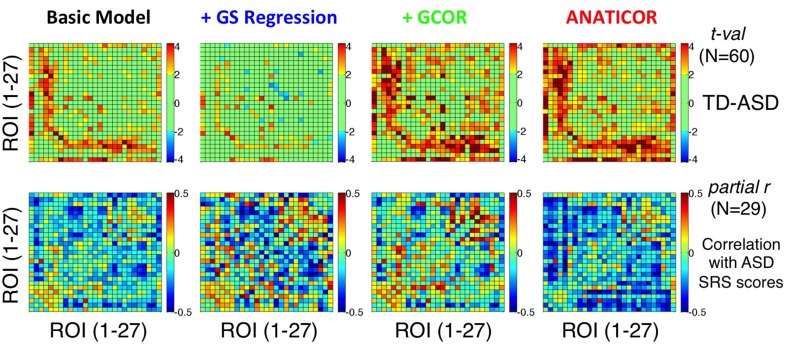
**Effect of preprocessing model on the agreement of group differences and social symptom correlations within the ASD group**. Group *t*-tests are shown for the four preprocessing models in the top row using ROIs 1–27 (Figure 3) (see colorbar for scale of *t*-values to the right). Partial correlations of SRS total score with ROI-ROI correlation level within the ASD group, removing shared variation with Age and full scale IQ, are shown in the bottom row (see colorbar for scale of partial *r*-values to the right). Only the ANATICOR model produced significant correspondence between the group differences and behavioral correlations solely within the ASD group (see text for details). The +GS model also failed to exhibit strong group differences using these ROIs, consistent with the results of Figure 6C.

One critical demonstration of our prior study (Gotts et al., 2012) is that the brain locations showing the largest group differences between ASD and TD groups are also those that exhibit the largest associations between correlation level and the severity of social impairment within the ASD group (indexed by SRS total score). In particular, among the 3 clusters of ROIs that we examined, the largest effects of both types (group differences and SRS correlations) occurred between the limbic-related ROIs of Cluster 1 (ROIs 1–7) and the remaining social brain regions in Clusters 2 and 3 (ROIs 8–27). In the current study, we evaluated the agreement of the group differences and SRS correlations for the four preprocessing models using these same 27 ROIs (see Figure 3). Results are presented in Figure 10, with group differences (*t*-tests: TD-ASD) shown in the top row and correlation with SRS total score, partialling Age and IQ (as in our previous study), shown in the bottom row. Symptom correlations in the case of the +GCOR model were conducted using the participant-specific correlation matrices under the Basic model, partialling the GCOR value for each participant along with Age and IQ. Yellow/red colors for the group comparisons indicate greater correlations for the TD group, blue colors indicate greater correlations for the ASD group, and light green indicates *t*-values that fail to reach a two-tailed significance level of *p* < 0.05. For the correlations with SRS within the ASD group, blue colors indicate that low ROI-ROI correlation levels predict high SRS total scores (i.e., lower correlation → higher social impairment), whereas yellow/red colors indicate the opposite relationship. Figure 10 shows that the locations of the strongest group differences (TD > ASD) are quite similar for the Basic, +GCOR, and ANATICOR models (between ROIs 1–7 and ROIs 8–27), while the +GS model shows weak or non-significant differences in these same locations. The relative lack of results in these locations is in agreement with the earlier results reported for Prediction 3 and shown in Figures 6C, 8.

On visual examination, the only preprocessing model of the four that exhibited good agreement between the group comparisons and symptom correlations was the ANATICOR model. This was examined in more detail statistically with the use of permutation tests (e.g., Maris and Oostenveld, 2007), as the column/row interdependencies of the matrices prevented easy estimation of the appropriate degrees of freedom. The quantitative agreement between the matrices in the top and bottom rows for each model was first assessed using Pearson correlation. Rather than using the *t*-values in the top row directly for these analyses, the group mean difference of the correlation values (TD-ASD) was used so that the same type of measure (with the same numerical scale/distribution) was being associated in both matrix types. After calculation of the *r*-values for the group difference and behavioral correlation matrices using the original data, *P*-values were estimated empirically by randomly re-labeling participants as either ASD or TD. The group comparisons and behavioral correlations were re-calculated for these randomly formed groups along with the corresponding Pearson *r*-value between matrices, and the entire randomization process was repeated 1000 times. The *P*-value (Type I error) for the original matrix agreement measures corresponded to the percentage of random iterations with an agreement value stronger than that observed for the original data. The Pearson *r*-values (and *P*-values) for the Basic, +GS, +GCOR, and ANATICOR models were −0.0477 (*p* > 0.3), −0.0198 (*p* > 0.4), −0.1051 (*p* > 0.1), and −0.2777 (*p* < 0.027), respectively. The significant negative *r*-value for the ANATICOR model indicates that ROI pairs with group differences favoring the TD group tended to be the same ROI pairs as those with a significant negative correlation with SRS score, as originally reported (Gotts et al., 2012).

**Figure 11:**
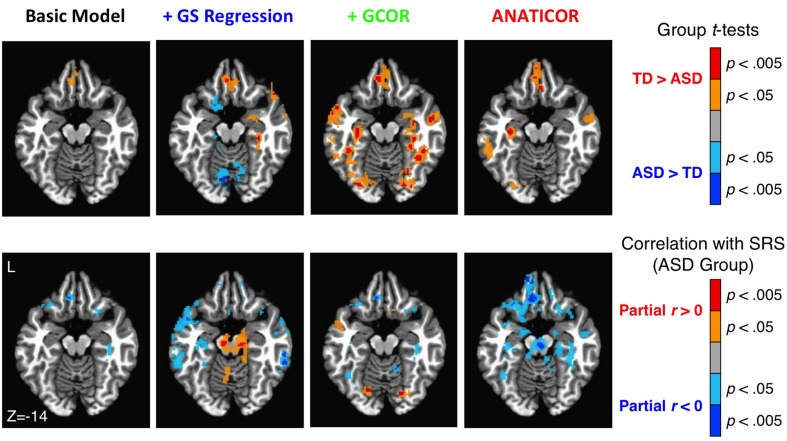
**Effect of preprocessing model on the agreement of group differences and ASD social symptom correlations using whole-brain connectedness**. Whole-brain connectedness was compared between groups for each of the four preprocessing models (top row; see colorbar to right for scale and direction of effects). Whole-brain connectedness for the ASD participants was also correlated with SRS total score, partialling Age and IQ, for the four models (bottom row; see colorbar to the right for scale and direction of effects). While select locations overlapped between the two effects for the Basic and +GCOR models, the best correspondence was still obtained under the ANATICOR model. The two effects were robust individually under the +GS model, but they exhibited little spatial overlap with each other and only minor overlap with the effects under the other models (e.g., TD > ASD in the ventromedial prefrontal cortex). Only the +GS model exhibited prominent reversed effects (ASD > TD) for the group comparisons (see also Figure 8).

The anatomical agreement of the group differences and symptom correlations could also be examined in a whole-brain fashion using the voxel-wise whole-brain connectedness values for each participant under the four preprocessing models (see also Figure 8 in the current paper; Figures 2, 5 in Gotts et al., 2012). These results are shown in Figure 11 using a single axial slice that captures the largest overlap of the two effects for the ANATICOR model (*z* = −14). As previously reported, the ANATICOR model shows a good agreement between the two effects, with spatial overlap of the results in three out of seven of the Cluster 1 ROIs (ROIs 1–7). Isolated locations of overlap between the two effects also exist for the Basic and +GCOR models (e.g., in the ventromedial prefrontal cortex), although the overall strength of the SRS correlations is notably weaker for these models. Group differences and SRS correlations were both robust when using whole-brain connectedness with the +GS model. However, they had little or no spatial overlap with one another. Furthermore, it was not just the group comparisons that were altered by GS regression relative to the other models: the SRS correlations solely within the ASD group were also strongly altered. This last effect underscores the point that the warping effect of GS regression on correlation matrices can be just as problematic for analyses involving single groups of participants.

## Discussion

The main goal of the current paper was to examine several theoretically motivated predictions regarding the detrimental impact of GS regression on group comparisons of functional connectivity. We tested these predictions in our previously published resting-state data of ASD and TD participants relative to three other preprocessing models of interest, including our original ANATICOR approach and a novel alternative to GS regression that we refer to as GCOR (see also Saad et al., 2013). In summary, we have demonstrated the following points:

1. GS regression does not simply re-center and/or re-scale a matrix of correlation values. It “warps”the values differentially in different voxel/ROI pairs as a function of the initial covariance matrix (see also Saad et al., 2012, 2013). This effect is not small. It reduced the Spearman rank correlations of matrices pre- and post-GS regression to approximately 0.7, sharing around 50% of the variance. This was not simply a function of removing unwanted global artifacts that influence BOLD fMRI. Results from two alternative methods, our ANATICOR approach that models physiological nuisance signals more explicitly and GCOR that partials the influence of the global level of correlation from the single-participant correlation values, were not altered comparably, with Spearman rank correlations of approximately 0.9 or higher (sharing better than 80% of the variance). The distortion of correlation values under GS regression was also well predicted by our prior mathematical analyses (correlations above 0.97 and 95% of variance accounted for), despite the fact that signals were not included from nuisance brain tissue compartments (white matter, ventricles, sinuses). Given that the distortion is a *systematic* function of the initial covariance matrix, results are expected to replicate well across labs and studies. In fact, the use of GS regression does increase the consistency of correlation estimates and correlation differences due to a reduction in brain-wide noise sources when such sources are otherwise unaccounted for (e.g., Fox et al., 2009; Keller et al., 2013). However this increased consistency is not a justification for the use of GS regression because it comes at the cost of rendering contrasts between groups with differing correlation structures uninterpretable, as illustrated in theory (Saad et al., 2012, 2013) and in practice here. Even single-group results will become distorted with GS regression (see bottom panels of Figures 10, 11).
2. GS regression substantially increases the number of locations that demonstrate greater correlation for ASD relative to TD participants, both long-range and local. Indeed, under GS regression the average *t*-value observed across all regions sampled (*N* = 1880) was significantly different from zero, favoring the ASD group. Greater correlations for the ASD relative to the TD group were not prominently observed in any of the other preprocessing models. Furthermore, the two alternative preprocessing methods that address more global artifacts (ANATICOR, GCOR) produced qualitatively similar results to one another, with reasonable agreement about the direction of effects and regions involved as in another recent whole-brain study of functional connectivity in ASD that did not apply GS regression (e.g., Anderson et al., 2011b). The locations involved also agree well with task-based studies of evoked responses in ASD and TD participants that employ social and linguistic stimuli (e.g., Castelli et al., 2002; Just et al., 2004; Di Martino et al., 2009; Kaiser et al., 2010; Lombardo et al., 2010; Dinstein et al., 2011; Weisberg et al., 2012). In contrast, a recent whole-brain resting-state study of ASD that applied GS regression found a mixture of increased and decreased correlations relative to TD participants (Rudie et al., 2013). Indeed, the negative correlations present under GS regression for both groups led to separate effects in both directions for the positive versus the negative correlations. Given the results reported in the current paper, it is not clear whether the greater correlations observed for the ASD group in the Rudie et al. study are real or produced by GS regression.
3. GS regression has a tendency to distort group comparisons most in locations that exhibit the strongest effects under other preprocessing models. ROIs that elicited the largest average seed-based correlation differences were the same ROIs for which the average correlation differences were most attenuated under GS regression (Figure 6C). When examining correlation differences among the 27 ROIs that yielded the largest effects in our prior study (Gotts et al., 2012), group differences were also mostly non-significant after GS regression (Figure 10). As discussed in the introduction, this occurs for a relatively simple mathematical reason: whole-brain connectedness is a direct function of the fit to the GS. The process of GS regression is to subtract this portion of variation from the results. This is not to say that all studies that employ GS regression will fail to find group differences similar to what we report here for ANATICOR or GCOR. Indeed, we already know from functional connectivity studies using a more restricted number of seed locations that a similar subset of results can be obtained when using GS regression (e.g., Kennedy and Courchesne, 2008; Ebisch et al., 2011; von dem Hagen et al., 2012; see Di Martino et al., 2013, for related discussion). However, we would expect such results to be larger in amplitude if an alternative such as GCOR were used, and we would also expect convergence toward the pattern of mixed increases and decreases if more seed locations are used.
4. Locations exhibiting group differences and ASD social symptom correlations no longer overlap with one another after GS regression. Using the matrix of 27 ROIs from our previous study to assess the quantitative agreement of these two effects, the correlation was near zero after GS regression, whereas there was a significant level of agreement using the ANATICOR model (*r* = −0.277, *p* < 0.03) (Figures 10, 11).

Taken together, our results strongly argue against using GS regression when comparing correlation values between groups of participants. It is difficult to avoid the conclusion that nothing can be demonstrated unequivocally about either the location or direction of group differences when this form of “de-noising” is applied. Given the further alteration of the SRS correlations solely within the ASD group (Figures 10, 11), it is clear that the “warping” effects of GS regression on individual correlation matrices may also affect results obtained within single groups of participants (e.g., whole-brain parcellations of functional areas/networks that utilize correlation measures). It may therefore be prudent to re-examine such results with an alternative approach, perhaps with GCOR or preferably with de-noising approaches that avoid signals from the gray matter regions of interest (e.g., Jo et al., 2010; Anderson et al., 2011a).

### Is there ever a legitimate reason to apply GS regression?

While our conclusions here regarding GS regression are quite negative, we would like to emphasize that there are good reasons for examining—and perhaps removing—global fluctuations in fMRI time series. In many respects, the Basic preprocessing model produced highly similar matrices to those produced by ANATICOR and GCOR; the Spearman rank correlations are all above 0.9 for both the average matrices and those of individual participants. However, the group comparisons using the Basic model failed to yield robust results, and it is worth considering why this occurred. The effect of removing global sources of variation, either by modeling physiological variation directly (ANATICOR) or partialling out the influence of the global level of correlation (GCOR), was not primarily to modulate the average correlation values for the ASD and TD groups (see results related to Prediction 1). Rather, the larger impact appeared to be on the variation across participants for a given pair of ROIs (as in Figure 4, top panel). A relatively small number of participants have large global levels of correlation in both groups (Figure 4, bottom panel), which when comparing the two groups has the effect of making the standard deviations that contribute to the denominator of the *t*-values large and thus the *t*-values themselves become small and non-significant. Attenuating the variation in each group then has the effect of shifting all of the *t*-values to be more positive (Figure 6A). Indeed, this is one of the primary motivations for applying GS regression, and it demonstrates that if global artifacts in the data are not modeled and removed sufficiently, then one will be at risk of making Type II statistical errors.

One possible example of doing too little to remove global artifacts is provided in the recent study by Tyszka et al. (2013). These authors did an admirable job of assessing the impact of head motion on group differences, which is one source of global artifact in fMRI time series. They found mostly weak and non-significant group differences between ASD and TD participants, smaller on average than the influence of high versus low levels of head motion. This led them to conclude that resting-state correlations in ASD participants are largely typical. However, no aspect of the preprocessing in this study directly addressed global artifacts other than head motion, and GS regression was not applied. Physiological variation will not typically be well removed by the popular bandpass filtering step (Tyszka et al., 2013, removed independent components with more than 33% of spectral power above 0.1 Hz), since the problematic frequencies (~0.3 Hz for respiration cycles and ~0.9–1 Hz for cardiac cycles) have already been aliased to frequencies below the Nyquist frequency (0.25 Hz in Tyszka et al. for *TR* = 2 s). Slower fluctuations in the BOLD response that result from spontaneous breath withholding during fMRI scans, due to end-tidal CO_2_ effects on BOLD measurements (Chang and Glover, 2009) and that are modeled by our RVT regressors (Birn et al., 2008), can have quite a large impact on resting-state correlations (>20–30% of total variance in some of our participants; see Supplementary Figure 1, Gotts et al., 2012). Since RVT regressors have most of their power (>90%) in frequencies below 0.1 Hz, bandpass filtering below 0.1 Hz will also fail to address this source of variation. Overall, one expects a preprocessing pipeline that does not address more global physiological artifacts to fail to find strong group differences, as shown in the supplement to our original paper (Gotts et al., 2012) and in the Basic model of the current paper (using regressors for motion, ventricles, and local white matter). In that sense, the results of Tyszka et al. (2013) are exactly in accord with our expectations. It would be quite useful to re-examine their results with a method such as GCOR to establish whether the *t-values* would shift to be more positive and significant as in our current study. Hardware artifacts, addressed by the local white matter regressor in all of the models in the current study (see Jo et al., 2010, 2013 for discussion), are another source of relatively global artifact that has received much less attention than merited. We agree with advocates of GS regression that removing the GS will be expected to attenuate all of these more global artifacts in the data, leading to stronger group differences, higher reliability of results, etc. This is the case for our current results relative to the Basic model. However, it will do so at the high cost of warping the matrices of interest, preventing any straightforward conclusions about group comparisons. Therefore, we cannot recommend its application, especially when cleaner alternative methods exist for removing global artifacts—including the distant-dependent effects of transient head motion documented by Power et al. (2012) and addressed recently in Jo et al. (2013).

### GCOR as an alternative to GS regression

The GCOR model yielded a pattern of group differences that largely replicated what we reported originally for ANATICOR. The strongest group differences were between limbic-related (ROIs 1–7) and non-limbic social brain regions (ROIs 8-27) (Figures 8, 10, 11). If anything, the group comparisons under GCOR were larger in magnitude. However, it failed to replicate the correlations with SRS score within the ASD group. This failure was not entirely unanticipated, since the approach explicitly alters the variation in individual correlation values around the mean (or median), which is the same as the primary measure used for the SRS correlations. Under the GCOR approach, there is no *a priori* way to correctly partition the global level of correlation into different sources, some of which should be removed (global artifacts such as head motion, physiological and hardware artifacts) and some of which should not (neurally generated global variation; e.g., Schölvinck et al., 2010). It is this issue that prevents us from enthusiastically endorsing it for general use as a covariate. However, its good performance for the group comparisons in the current study suggests that it may be useful for conducting group comparisons in seed-based correlation studies when physiological de-noising is not possible due to lack of cardiac and respiration measures. It is also useful as a diagnostic tool to assess the distribution of global correlation levels in different groups. With subsequent work on this and other forms of data standardization (e.g., Yan et al., 2013), a post-hoc correction that works well for both group comparisons and symptom correlations may eventually be discovered, preserving datasets that were acquired without independent physiological measures. However, any time that nuisance measures are taken from the data that they are intended to clean, the risk is high for collinearity with the grouping variable, necessarily leading to GS-regression-like effects to some degree (Saad et al., 2013). It will be essential to examine any such methods with both simulations and mathematical analyses for the biases that they can introduce into single- and multi-group analyses. For example, in the current GCOR method, it is critical to examine the issue of data centering and to verify that the distributions of the covariates in the two groups are substantially overlapping (see Figure 4). If covariate distributions are non-overlapping in the two groups and a single grand mean center for the covariate is used, GCOR will introduce distortions similar to those introduced by GS regression (Saad et al., 2013), although such distortions will likely fail to reach significance because the covariate is highly collinear with the grouping variable. If group-specific centering is chosen (as in the current study), then it is possible that the difference in average correlation between the two groups is based on an artifactual source of global variation rather than a real neural difference. The best current alternative is to collect independent measures of physiological variation, modeling their influences separately. Given the impact that these preprocessing choices can have on the results that one obtains, it is difficult to overstate the importance of collecting heart rate and respiratory waveforms at the time of data acquisition.

### Are studies of functional connectivity doomed by artifacts?

One reaction to the data that we have presented is that the pattern of data one finds is strongly influenced by choice of preprocessing model. Without knowing which model is the correct one to use, how can we be confident in any of the results? Our reaction to the data is more optimistic than that. For three of the models examined (Basic, GCOR, and ANATICOR), the overall ROI-ROI structure of the correlation matrices and group comparisons was remarkably similar across models. One would not be led down a substantially different theoretical pathway by either of the two non-GS models that address global artifacts. Instead, our results highlight the importance of addressing *all* of the major classes of time-varying artifacts that MR methods research has identified for BOLD fMRI (i.e., head motion, physiological, and hardware). In principle, many preprocessing models - including ICA-based models, can do a sufficient job at addressing this family of artifacts. We view certain alternatives to standard regression-based data cleaning, such as multi-echo fMRI with ICA to sort BOLD from non-BOLD variation (Kundu et al., 2012), as extremely promising. Future studies comparing such alternatives with single-echo fMRI that is acquired along with independent physiological measures should help to clarify which data-cleaning approaches work the best.

### Conflict of interest statement

The authors declare that the research was conducted in the absence of any commercial or financial relationships that could be construed as a potential conflict of interest.
